# Feature tracking strain analysis detects the onset of regional diastolic dysfunction in territories with acute myocardial injury induced by transthoracic electrical interventions

**DOI:** 10.1038/s41598-022-24199-1

**Published:** 2022-11-14

**Authors:** Kady Fischer, Philipp Becker, François-Pierre Mongeon, Janelle Yu, Gobinath Nadeshalingam, Matthias G. Friedrich, Dominik P. Guensch

**Affiliations:** 1 Philippa and Marvin Carsley CMR Centre at the Montreal Heart Institute, Montreal, QC Canada; 2 Department of Anaesthesiology and Pain Medicine, Inselspital, Bern University Hospital, University of Bern, Bern, Switzerland; 3 Department of Diagnostic, Interventional and Paediatric Radiology, Inselspital, Bern University Hospital, University of Bern, Bern, Switzerland; 4 Department of Medicine, McGill University, Montreal, QC Canada; 5 Department of Radiology, McGill University, Montreal, QC Canada

**Keywords:** Cardiology, Experimental models of disease, Magnetic resonance imaging

## Abstract

Electric interventions are used to terminate arrhythmia. However, myocardial injury from the electrical intervention can follow unique pathways and it is unknown how this affects regional ventricular function. This study investigated the impact of transthoracic electrical shocks on systolic and diastolic myocardial deformation. Ten healthy anaesthetized female swine received five transthoracic shocks (5 × 200 J) and six controls underwent a cardiovascular magnetic resonance exam prior to and 5 h after the intervention. Serial transthoracic shocks led to a global reduction in both left (LV, − 15.6 ± 3.3% to − 13.0 ± 3.6%, p < 0.01) and right ventricular (RV, − 16.1 ± 2.3% to − 12.8 ± 4.2%, p = 0.03) peak circumferential strain as a marker of systolic function and to a decrease in LV early diastolic strain rate (1.19 ± 0.35/s to 0.95 ± 0.37/s, p = 0.02), assessed by feature tracking analysis. The extent of myocardial edema (ΔT1) was related to an aggravation of regional LV and RV diastolic dysfunction, whereas only RV systolic function was regionally associated with an increase in T1. In conclusion, serial transthoracic shocks in a healthy swine model attenuate biventricular systolic function, but it is the acute development of regional diastolic dysfunction that is associated with the onset of colocalized myocardial edema.

## Introduction

Atrial fibrillation is the most common sustained cardiac arrythmia worldwide, and is estimated to occur in approximately one-third of individuals^1^. A key procedure to restore the heart’s normal rhythm in both immediate life-threatening arrhythmias and in elective situations are electrical interventions, defibrillation and cardioversion. Defibrillating shocks are used in emergency situations to terminate ventricular fibrillation or in the presence of unstable hemodynamics during ventricular tachycardia. Otherwise, cardioversion, applying R wave-triggered synchronized shocks, is the process of converting patients with atrial fibrillation back into sinus rhythm. External electrical cardioversion was first successfully performed in the 1950s^2^. Despite more than 70 years of using the technique, the effects of electrical interventions are still unclear. We previously showed using cardiovascular magnetic resonance (CMR) imaging that serial transthoracic shocks resulted in acute myocardial edema^3^. This was measured by increased myocardial T1, which was further confirmed histologically. Edema is often featured in acute myocardial injury, and can be identified with native myocardial T1 mapping^4,5^. However, myocardial injury follows unique pathways through the thorax in each individual and it is unknown how it impacts regional ventricular function. In swine, Aeillo et al. demonstrated that increased electrical burden, defined by the cumulative energy of unsuccessful shocks, was linked to reduced LV function and survival^6^. Common functional measures such as ejection fraction (EF) and volumes are limited in that they represent only global ventricular function and the effects of transthoracic shocks on regional myocardial function have yet not been systematically assessed.

**Figure 1:**
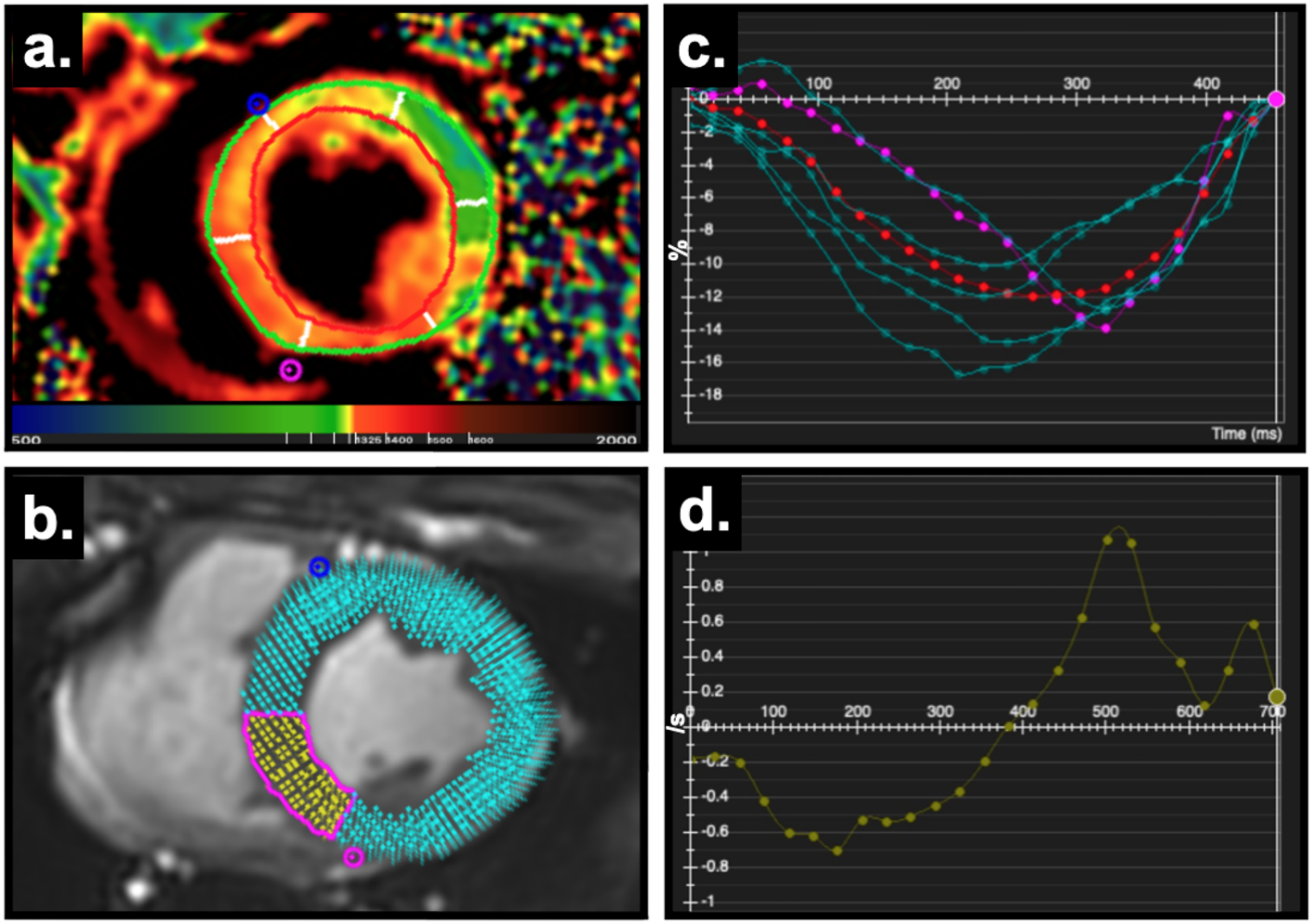
CMR as a non-invasive tool for assessing regional tissue, systolic and diastolic abnormalities. (**a**) T1 map with the left ventricle segmented into six territories; orange depicts higher T1 after the intervention, (**b**) feature tracking strain highlighting the inferoseptal segment, (**c**) circumferential strain curve of each of the six segments displaying mechanical dispersion after the electrical intervention (pink highlighted inferoseptal segment in (**b**), red is the global strain curve) and (**d**) strain rate curve.

CMR feature tracking (FT) is a post-processing technique that can quantify myocardial contraction and relaxation on a regional level (Fig. 1) and can detect subtle changes in function for both the left and right ventricle^7^. Because strain can detect subtle abnormalities, even in the absence of global systolic impairment, it is thus interpreted as an earlier and more sensitive modality than conventional visual assessment. Multiple publications have reported that CMR-FT incrementally improves diagnostics and prognostication beyond the common ventricular function measures such as ejection fraction^8–11^. Strain has also been used for repetitive measures within the same subject to investigate temporal changes in function occurring over a short period of time^12–15^. In addition to assessing regional function, strain analysis is beneficial as multiple markers representing systolic and diastolic function can be derived simultaneously. This multiparametric approach of strain measurements is beneficial as it provides a comprehensive assessment of various components of myocardial function over the cardiac cycle^16,17^. CMR-FT has also been translated to experimental animal models^15,18^, and has been reported to be reproducible in swine hearts^19^, and related to myocardial injury^20,21^.

The purpose of this study was to investigate the impact of acute myocardial injury after serial transthoracic electrical shocks on regional systolic and diastolic myocardial deformation in a healthy animal model.

## Methods

### Ethical statement and anaesthesia

The study was performed in accordance with the ARRIVE guidelines and with the “Guide to the Care and Use of Experimental Animals” by the Canadian Council on Animal Care and approved by the animal care board of the Montreal Heart Institute, Montreal, Canada [#20125003]^3^ prior to the start of the study. Seventeen female common landrace swine (32 ± 1 kg body weight), were premedicated with combination of 200 mg zolazepam, 200 mg tiletamine. 0.8 mg atropine and 2–4 mg/kg of intravenous propofol induced anaesthesia. Animals were then intubated and mechanically ventilated for the remainder of the experiment. A continuous propofol infusion (12–37 mg/kg/h) maintained anesthesia. For blood sampling, invasive blood pressure monitoring, as well fluid infusion, the femoral artery and vein were cannulated. At the end of the imaging experiment 40 mmol of intravenous KCL and 200 mg propofol was used for euthanasia.

### Experimental protocol and image acquisition

The animals were transferred to a clinical 3 Tesla MRI (Magnetom Skyra, Siemens Healthineers AG, Erlangen Germany). Prior to any intervention all animals underwent a baseline imaging protocol involving native T1 maps acquired in three short axis views (basal, mid-ventricular and apical) and a short-axis cine stack of 7–10 slices crossing the ventricles. A 3(3)3(3)5-modified Look-Locker sequence was used for the T1 imaging (temporal resolution/echo-time: 2.6 ms/1.08 ms, flip angle 35°, voxel size 1.6 × 1.6 × 8.0 mm, bandwidth 1085 Hz/Px). An ECG-gated balanced steady-state free precession sequence was used for the cine imaging (temporal resolution/echo-time: 1.43 ms/3.3 ms, flip angle 65°, voxel size 1.6 × 1.6 × 6.0 mm, matrix 192 × 120, bandwidth 962 Hz/Px, 25 phases).

**Figure 2:**
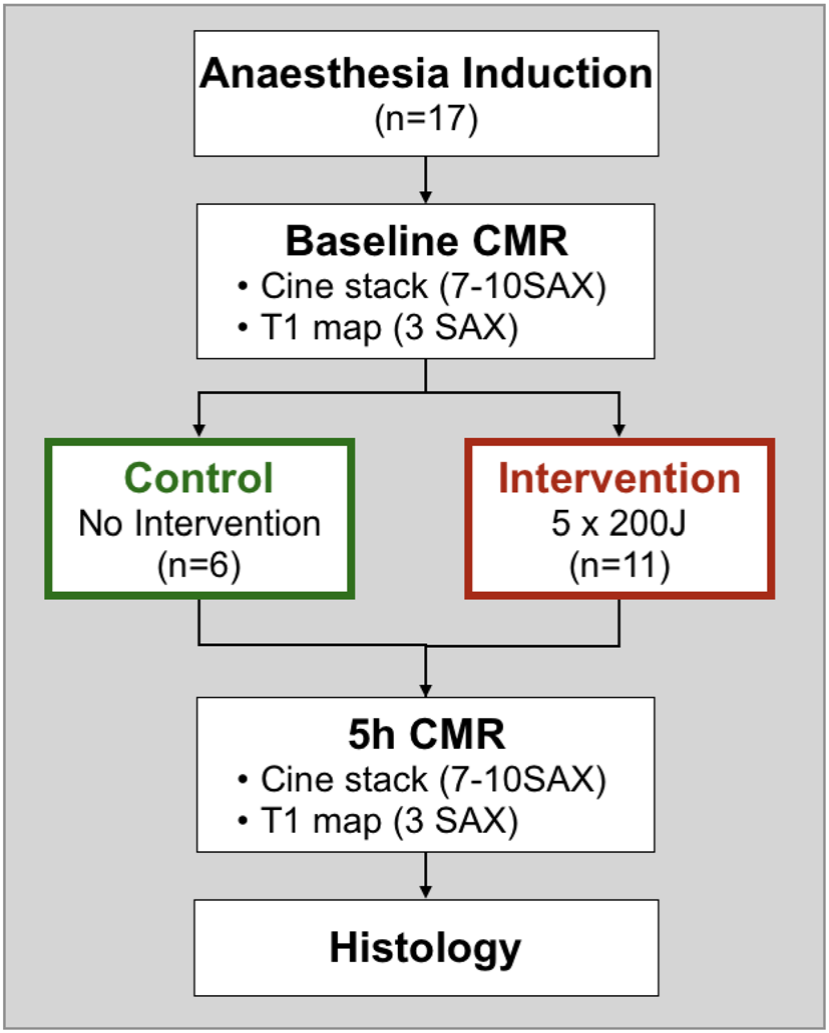
Study protocol. *CMR* cardiovascular magnetic resonance exam, *SAX* short axis stack.

Six animals served as controls, while eleven underwent the electrical intervention. For this group one defibrillator pad was placed on the left postero-lateral chest wall in front of the scapula, and the second was placed on the right pectoral muscle close to the axilla. Five serial biphasic synchronized transthoracic shocks were applied using an automated external defibrillator (LifePeak 15 Defibrillator, Physio Control, Inc. Redmond, WA, USA). Five hours later, all subjects underwent cine and T1 imaging, similar to the baseline exam (Fig. 2).

### CMR image analysis

All datasets were recoded to blind the readers to the intervention, and one reader quantified T1 maps for both the LV and RV. T1 analysis of the LV was performed on the generated pixel-wise maps with placement epicardial and endocardial contours on 3 short axis slices, and data were reported for each of the 6 myocardial segments on the basal, mid and apical slices (18-segment model, Fig. 1). For RV T1 analysis, a region of interest was contoured across the RV inferior and lateral wall of each of the 3 short-axis slices. Due to the thin RV myocardium and free wall motion, contours were drawn on each of the individual raw T1-weighted images to avoid the limitations associated with reconstruction of an in-line pixelwise relaxation map, and a T1 relaxation curve was generated by the software from the individual region of interest contours.

A second reader blinded to the tissue characterization findings analyzed biventricular ejection fraction and deformation on the short-axis stack. For the FT strain measurements, the parameters of circumferential peak strain, time to peak strain and early diastolic strain rate were acquired for global measurements of the LV myocardium and RV free wall. To measure the regional response in LV FT strain measures, data were acquired per segment in the slices matching the T1 analysis. Mechanical dispersion was calculated as the standard deviation of the time to peak strain of all segments. For regional analysis of the RV, each acquired slice (Fig. 5) was considered as an independent region. All image analysis was performed using cvi^42^ (Circle Cardiovascular Imaging, Calgary, Canada).

### Histology and serology

After euthanasia, 34 samples from the LV were available for histological analysis, with regional selection either randomly selected or guided by T1 analysis. The fraction of interstitial space was quantified from three representative areas of each sample using semi-quantitative planimetry slice (ImageJ version 1.46, National Institute of Health, USA). Blood samples were acquired at baseline and the 5 h timepoint and troponin I (TnI), porcine cardiac fatty acid binding protein (cFABP), creatine kinase (CK) and porcine cardiac creatine kinase-MB (CK-MB) were quantified as detailed previously^3^.

### Statistical analysis

Initially, the difference in global CMR measures from baseline to the 5 h timepoint were compared for each group using a paired *t* test. The regional changes in strain in comparison to the development of myocardial injury (ΔT1 mapping) over the course of 5 h were statistically assessed using a mixed effects model accounting for the electrical intervention and multiple measurements per individual^15,17^. Finally, receiver operator characteristics curves were calculated to assess the strain markers’ ability to discriminate myocardial injury defined by histology and incremental cut-offs of intracellular area indicating increasing levels of tissue injury. Statistical significance was defined with a two-sided p-value of < 0.05. GraphPad Prism version 9.0 (GraphPad Software, La Jolla California USA), *R* software (version 3.5.0, R Foundation for Statistical Computing, Vienna, Austria) were used for analysis.

## Results

Ten swine receiving 5 serial transthoracic shocks of 200 J and 6 control animals successfully completed the protocol with CMR images repeated 5 h after the intervention (Fig. 2). One animal was excluded due to ventricular fibrillation during the electrical intervention with failure to be defibrillated. The animals had similar haemodynamics and blood sampling measurements at the baseline and the 5 h timepoint (Table 1). Fluid management with lactated Ringer’s solution (73 ± 22 vs. 73 ± 20 ml/kg, p > 0.99) and anaesthesia with propofol (20 ± 11 vs. 17 ± 11 mg/kg, p = 0.61) were similar for the control and shocked groups. Significant increases in CK, cFABP and CK-MB indicated tissue injury within the shocked group (Table 1), as reported previously^3^.

**Table 1:** Haemodynamics and blood samples.

	Controls			Shocked		
	Baseline	5 h	p	Baseline	5 h	p
**Haemodynamics**						
Heart rate (bpm)	100 ± 6	107 ± 7	0.20	98 ± 4	95 ± 6	0.89
Systolic blood pressure (mmHg)	91 ± 12	97 ± 14	0.25	88 ± 13	90 ± 12	0.52
Diastolic blood pressure (mmHg)	59 ± 9	63 ± 20	0.51	52 ± 10	57 ± 4	0.10
**Blood sampling**						
Haemoglobin (g/dl)	9.9 ± 0.9	9.0 ± 0.8	0.48	9.3 ± 2.2	8.7 ± 0.7	0.43
Haematocrit (%)	30.5 ± 2.8	29.4 ± 0.4	0.84	28.7 ± 6.7	28.7 ± 1.5	0.45
Troponin I (ng/ml)	0.5 [0.4–1.0]	1.1 [0.4–1.5]	0.50	0.5 [0.3–0.6]	0.4 [0.1–0.9]	0.80
cFABP (ng/ml)	6.9 [5.3–8.3]	6.5 [5.5–8.8]	0.69	8.0 [5.4–15.4]	14.6 [12.9–21.0]	0.01*
Creatine kinase (U/l)	650 [457–3149]	858 [423–2539]	0.69	602 [523–677]	7542 [5681–8802]	< 0.01*
Creatinine kinase MB (ng/ml)	0.45 [0.27–0.68]	0.65 [0.57–0.75]	0.06	0.55 [0.40–0.66]	0.66 [0.59–0.71]	0.03*

Mean ± SD or median [interquartile range] demonstrate haemodynamics and fluid status were maintained to a consistent level from baseline to the 5 h timepoint for each group, while the majority of serology markers increased in the shocked group only. *p < 0.05 represents a significant difference between baseline and 5 h measures. *cFABP* porcine cardiac fatty acid binding protein. Significant values are in bold.

### Change in global strain

The measurements of the global circumferential strain parameters at baseline and 5 h post-shock are depicted in Table 2. While there was no change in right (RV) or left ventricular (LV) ejection fraction for either group, global peak circumferential strain worsened in both ventricles in the shock group only (LV: − 15.6 ± 3.3% to − 13.0 ± 3.6%, p < 0.01; RV: − 16.1 ± 2.3% to − 12.8 ± 4.8%, p = 0.03). Additionally, LV mechanical dispersion was increased in the shocked group post-shock (p = 0.02). Through the experiment, LV early diastolic strain rate slowed from baseline to the 5 h timepoint (1.19 ± 0.35 to 0.95 ± 0.37/s, p = 0.02) in shocked animals. No change was observed for any global strain parameters in the control group.

**Table 2:** Global ventricular function parameters.

	Controls			Shocked		
	Baseline	5 h	p	Baseline	5 h	p
**Global LV measures**						
Ejection fraction (%)	58 ± 8	55 ± 10	0.30	58 ± 10	55 ± 8	0.26
Peak strain (%)	− 16.5 ± 0.8	− 15.0 ± 2.3	0.22	− 15.6 ± 3.3	− 13.0 ± 3.6	< 0.01*
Time to peak strain (ms)	292 ± 39	264 ± 32	0.27	299 ± 28	303 ± 56	0.88
Mechanical dispersion (ms)	45 [38–65]	36 [27–59]	0.31	42 [37–45]	56 [31–68]	0.02*
Early diastolic strain rate (/s)	1.11 ± 0.17	1.22 ± 0.19	0.30	1.19 ± 0.35	0.95 ± 0.37	0.02*
**Global RV measures**						
Ejection fraction (%)	48 ± 12	45 ± 10	0.46	48 ± 13	49 ± 15	0.64
Peak strain (%)	− 13.0 ± 3.6	− 12.4 ± 3.7	0.68	− 16.1 ± 2.3	− 12.8 ± 4.2	0.03*
Time to peak strain (ms)	284 ± 29	274 ± 26	0.55	291 ± 38	290 ± 27	0.99
Early diastolic strain rate (/s)	0.84 ± 0.20	0.94 ± 0.27	0.27	1.06 ± 0.22	0.93 ± 0.33	0.30

Mean ± SD, or median [interquartile range] are shown for global measures at baseline and the 5 h time point. *p < 0.05 represents a significant difference between baseline and 5 h measures. Significant values are in bold.

### Association of regional LV strain with tissue markers

**Figure 3:**
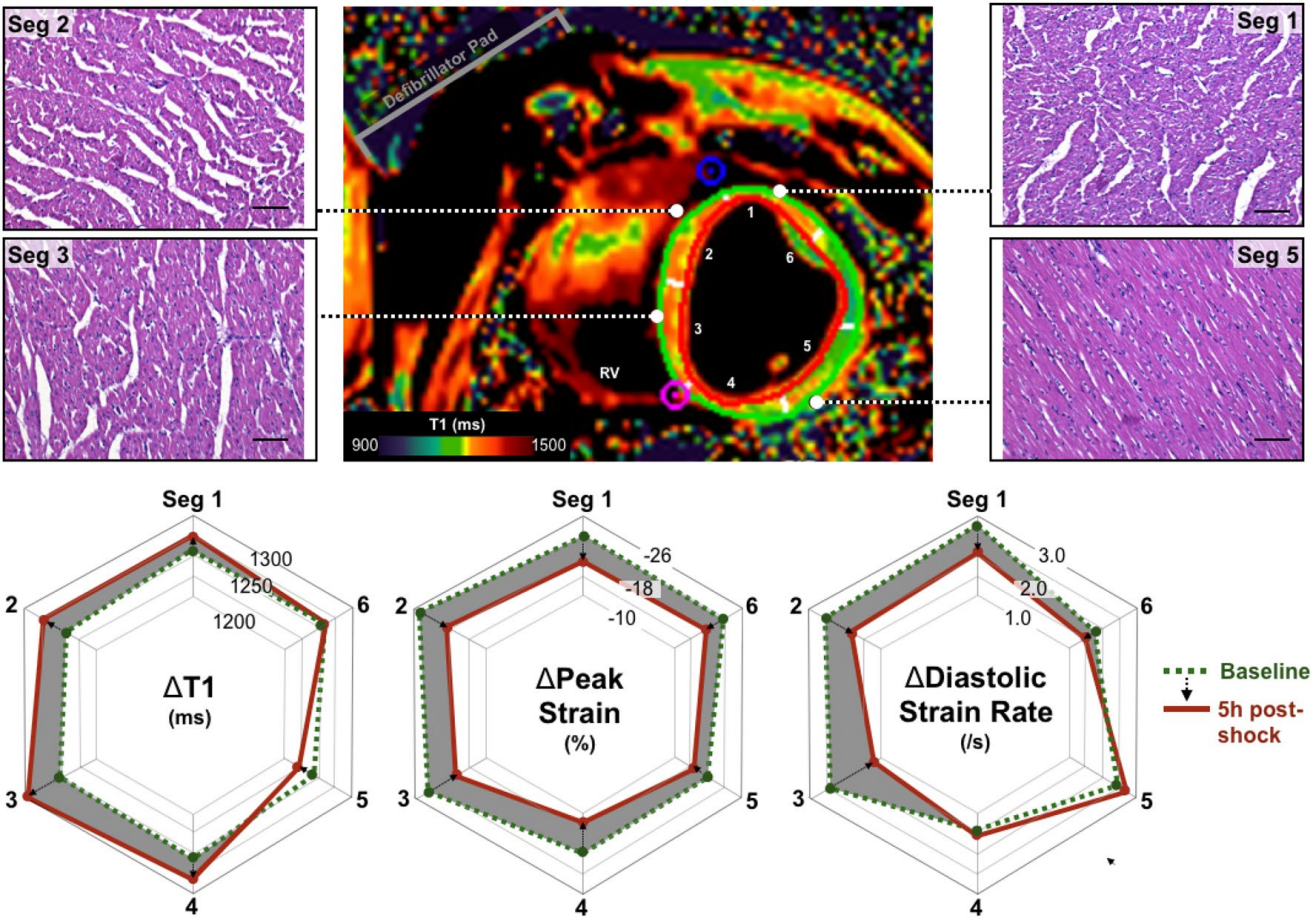
Regional impact of electrical interventions on the LV. For the respective slice, the T1 map acquired at 5 h post electrical intervention depicts higher signal (orange) in the septum indicating myocardial injury. This matches the histological samples in which interstitial space is greatest in sample acquired from the septum in comparison to the lateral wall. The polar plots on the bottom row depict the quantitative measurements for each of the six segments in the slice at baseline (green) and 5 h post intervention (red), with the change between timepoints shaded in grey.

As Figs. 1 and 3 demonstrate, the pattern of myocardial injury may follow a regional pathway and not impact the entire myocardium homogenously, leaving some areas of the myocardium unaffected. The change in LV T1 measurements, where images were acceptable for both timepoints was available for 267 segments (7% segmental exclusion rate). The linear relationship between LV regional function parameters and the increased regional tissue water content demonstrated by T1 are displayed in Table 3. There was no link between systolic peak strain and changes in T1. However, for systole, a prolongation in time to peak strain was observed with increases in T1 (p = 0.02). An increase in T1 was also correlated to a reduction in early diastolic strain rate (p = 0.03), where early diastolic strain rate slowed by − 0.25 s^−1^ for every 100 ms increase in T1.

**Table 3:** Linear relationship of regional parameters with ΔT1.

	ΔT1 (per 100 ms)	
	Beta	p-value
**Regional LV**		
ΔPeak strain (%)	0.02	0.97
ΔTime to peak strain (ms)	11.2	0.02*
ΔEarly diastolic strain rate (/s)	− 0.25	0.03*
**Regional RV**		
ΔPeak strain (%)	2.66	< 0.01*
ΔTime to peak strain (ms)	0.95	0.95
ΔEarly diastolic strain rate (/s)	− 0.42	< 0.01*

Linear relationship of regional function parameters in comparison regional changes in regional tissue water content demonstrated by T1 (per Δ100 ms) from the respective ventricle. *p < 0.05. Significant values are in bold.

**Figure 4:**
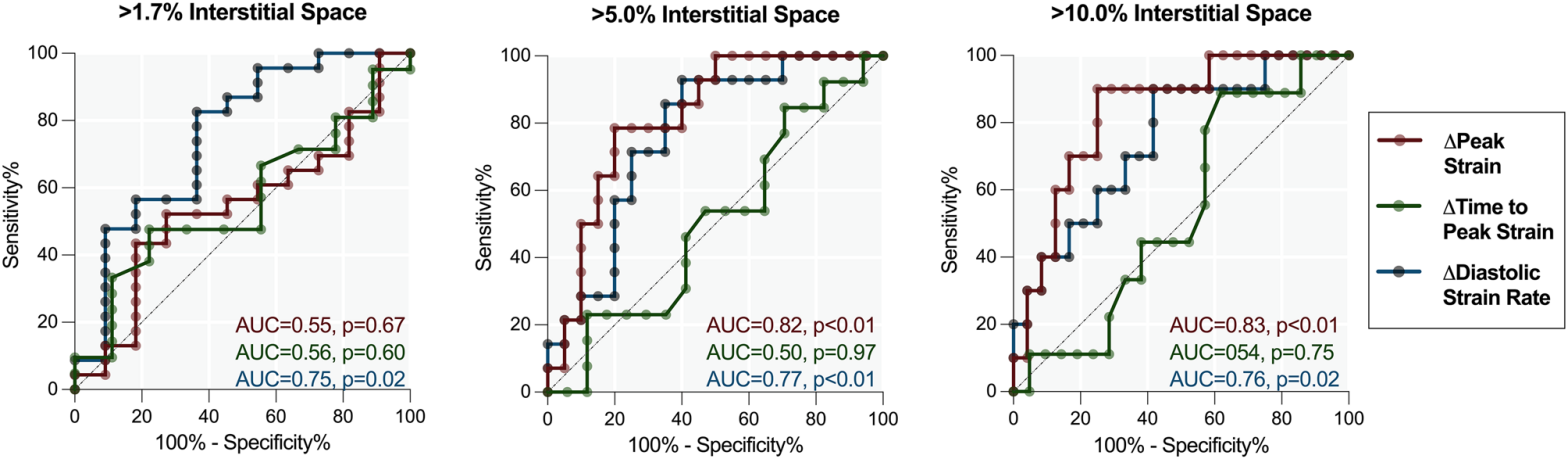
Ability of CMR feature tracking to discriminate incremental increases of LV interstitial space. Receiver operating curves demonstrate the area under the curve of the regional diastolic and systolic feature tracking strain measurements in comparison to localized samples assessed histologically for interstitial space. Analysis was performed using three cut-offs increasing with more severe myocardial injury.

Thirty-four LV histology samples were assessed for interstitial area (control: 1.1 ± 0.2%, shocked: 9.4 ± 1.7%) and localized to the segments matching the feature tracking analysis. When using a low cut-off for increased interstitial space, only diastolic strain rate was associated with mild myocardial injury (Fig. 4). Only in samples with moderate-severe (> 5%, and > 10%) interstitial space, was peak strain associated with histologically defined myocardial injury. No link was observed with time to peak strain. This can be visualized in a case example presented in Fig. 3. It can be observed that peak strain worsened homogenously across the myocardium, whereas T1 and histology samples show the bulk of the injury is localized across the septum and inferior wall which matches the regional variation observed in diastolic strain rate as well.

### Association of regional RV strain with tissue markers

**Figure 5:**
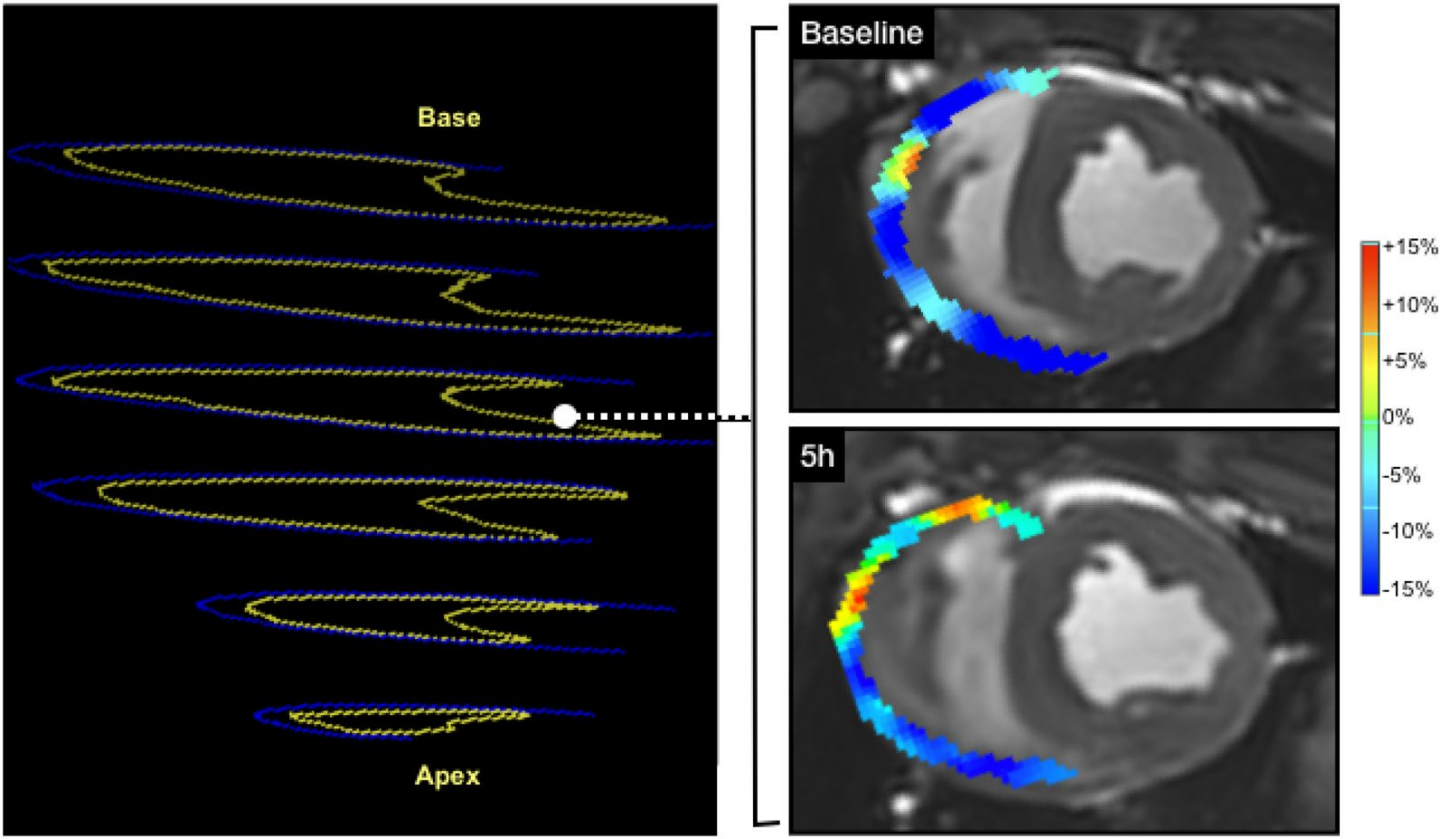
Impact of electrical interventions on the RV. Left: slice positions of the right ventricle (endocardium = yellow, epicardium = blue). Right: circumferential strain at end-systole indicating a worsening in strain (yellow-orange) 5 h after electrical intervention.

The change in RV T1 measurements, where images were acceptable for both timepoints was available for 40 slices (17% regional exclusion rate). Similar to the LV, RV diastolic function of an individual slice also worsened in the presence of increased RV T1, with a reduction of − 0.42 s^−1^ for every 100 ms increase in T1. Moreover, only in the RV was peak strain attenuated in relation to colocalized T1 (Table 3, Fig. 5).

## Discussion

**Figure 6:**
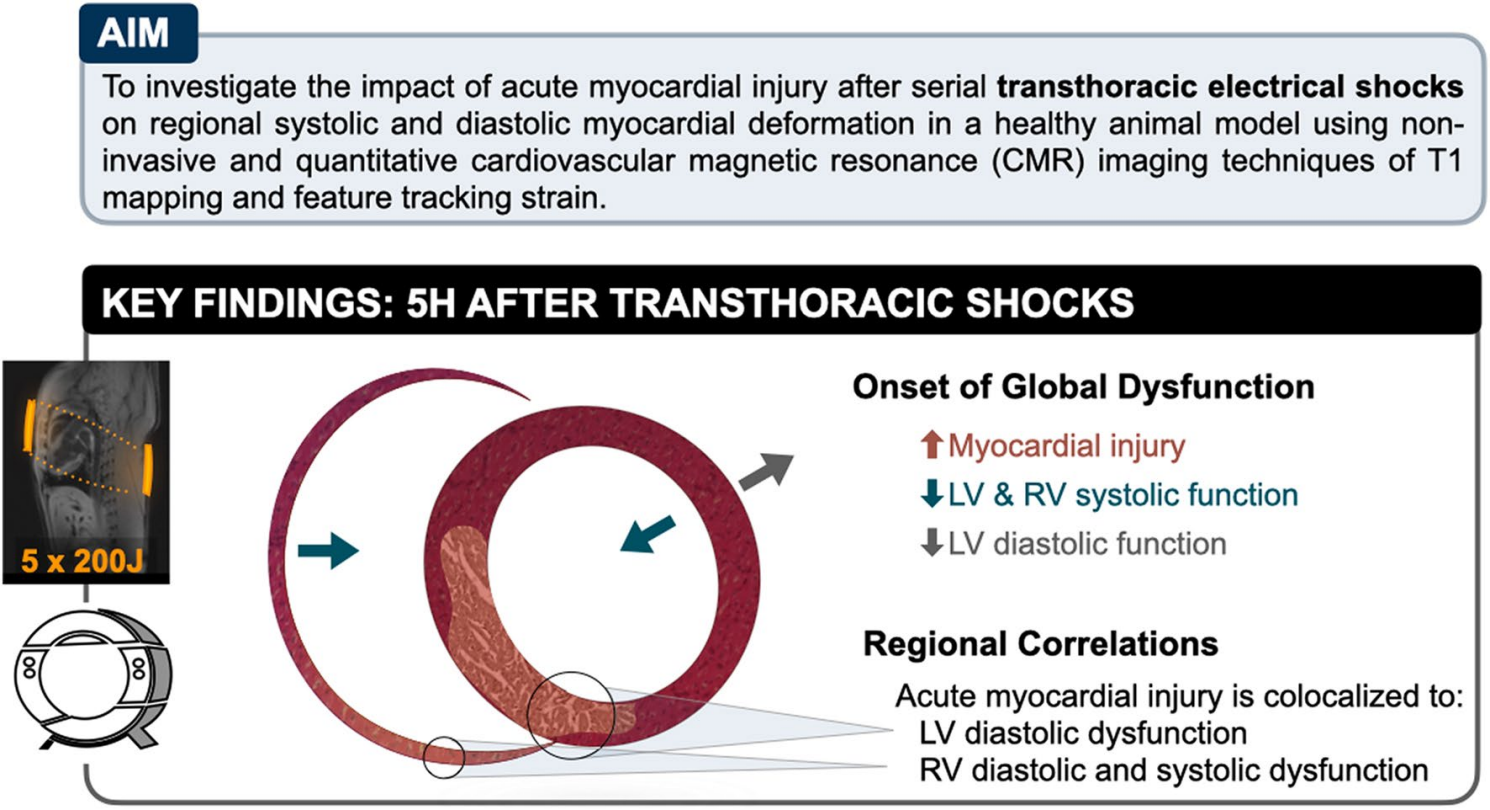
Summary figure. The illustration highlights the aim of the study and the key findings of global and regional systolic and diastolic dysfunction assessed by CMR feature tracking strain techniques, and of myocardial tissue injury quantified non-invasively by CMR T1 mapping and serology and by invasive histological assessments.

In a heathy swine model, serial transthoracic shocks led to a global reduction in both right and left ventricular systolic function within 5 h of the intervention assessed by circumferential strain analysis with CMR feature tracking (Fig. 6). This electrical intervention triggered the onset of regional myocardial edema in both ventricles observed by tissue characterization and increased interstitial space. We could additionally show that the extent of this injury was related to an aggravation of regional myocardial diastolic function in both ventricles. To the best of our knowledge this is the first published investigation about the onset of regional changes in systolic and diastolic biventricular function after transthoracic shocks. Previous imaging investigations have focused on global volumetric changes, while other non-invasive techniques have utilized serology markers^3,6,22,23^. Yet, the electrical pathway from transthoracic shocks can vary across the heart and these global techniques cannot identify the unique distributions of myocardial injury that occur as a result. The development of feature tracking analysis to quantify regional strain allowed us to colocalize the myocardial dysfunction with acute changes in myocardial tissue injury providing a more sensitive assessment of acute myocardial caused by electrical interventions.

### Acute myocardial injury

The myocardial injury due to electrical shocks has been theorized to be a consequence of either local heating to high temperatures or as a result of breakdown of the cell membrane caused by electroporation^24,25^. In electroporation injury, the applied current forces ions through the cellular membranes, which disrupts the delicate balance of ions and molecules along the intracellular and extracellular side of the cell membrane, therefore causing an osmotic water influx into the cell and edema^26^. Many studies have focused on serological markers, such as troponins and creatinine kinases^22,23,27^. However, the evidence is not consistent and biomarkers cannot determine the exact location of the injury. A more direct approach has been assessed through histology, although a key limitation is histological findings require an invasive biopsy or post-mortem analysis. Imaging provides an ideal non-invasive approach for investigation into regional and current myocardial injury, and repetitive acquisitions can be easily acquired to allow for monitoring dynamic changes in cardiovascular features over time. Especially with CMR, a comprehensive approach can be undertaken to monitor both myocardial injury defined in this study by increases in myocardial T1 from quantitative T1 mapping sequences, and coinciding changes in biventricular function. Particularly, quantitative approaches such as strain analysis, provide a sensitive investigation into functional changes. As our results depict, both systolic and diastolic strain parameters worsened globally in the intervention group, despite the fact that neither RV nor LV ejection fraction changed. These findings are reflected by multiple publications from echocardiography and magnetic resonance imaging modalities where it was reported that strain provides earlier or incremental information on myocardial function, especially in preserved ejection fraction cohorts, when no change in ejection fraction is noted, or even as an early predictor for changes in ejection fraction^17,28–33^. Although our findings demonstrate global dysfunction, strain allows an even deeper quantitative analysis into the regional variations of functional change, consequently it can also be colocalized to regional tissue markers.

### Diastolic dysfunction

Segmental LV diastolic strain rate was colocalized to both imaging and histologic markers of edema. While native T1 is also linked to myocardial fibrosis, a rapid increase in T1 over a short period of a few hours can be attributed to increased local water content associated with swelling. Moreover, an increase in interstitial space on histology could be due to electroporation injury of the smooth muscle vascular endothelium or through a subsequent inflammatory response. A deceleration in regional LV diastolic strain rate was linearly associated with the increase in myocardial T1, while no linear connection was observed with LV peak strain, a marker of systolic function. Additionally, LV diastolic strain rate could discriminate even mild levels of interstitial edema shown by interstitial area in histological analysis, while peak strain could only detect a greater severity of interstitial area. The aggravation of diastolic dysfunction in accordance with increased myocardial edema likely attributed to stiffening of the heart. When myocardial edema accumulates, the rise of interstitial pressure increases myocardial stiffness reducing ventricular compliance^17,34,35^. Detwiller showed in an isolated swine heart model that the stress–strain relationship of papillary muscles shifted with progressive edema, indicating increased stiffness^36^. However other mechanisms for edema-induced diastolic dysfunction have been proposed as well including, reduction of the myocardial oxygen supply through increased vascular resistance, abnormal extravascular pressure, and thicker myocardial walls^17,34,37^. Many of these mechanisms can impact contractility as well, with edema linked to both diastolic and systolic dysfunction^38^. Although we observed that systolic function worsened globally over the 5 h, we did not observe a direct regional correlation between systolic dysfunction and T1 in this study, and only with a significant interstitial area was systolic dysfunction locally associated with myocardial injury. As this analysis only investigated changes in edema and cardiac function over 5 h, this may indicate that diastolic dysfunction is impacted earlier than systolic function after the onset of myocardial edema in the LV. Interestingly, diastolic function being negatively affected prior to systolic function can also be seen in the well documented ischemic cascade^39^. The RV showed linear correlations with increased T1 and both peak circumferential strain and diastolic strain-rate, indicating that increased RV edema induced systolic and diastolic dysfunction during this timeframe. This could potentially be due to the fact the RV wall is thinner and may be more compromised by edema, or by the fact the RV could be more directly in the electrical pathway through the torso and in proximity to the defibrillator pad. Longer term studies would need to assess the temporal relationship between myocardial edema, and systolic and diastolic dysfunction. In a rabbit model it was shown that low-energy defibrillation shocks increase endothelin-1 levels both locally in the heart and in the blood serum, and the authors suggested this may lead to capillary blood flow abnormalities and potential post-intervention disease^40^.

### Right ventricular dysfunction

While the majority of investigations focus on the left ventricle, we undertook a biventricular analysis. The pathway of electrical interventions crosses the heart and is very likely to involve the RV which can be observed in Fig. 2. Yet RV affection from electrical interventions has not yet been systematically assessed. While tissue characterization has key advantages, it is limited in that standard sequences are not ideal for the assessment of the RV in most situations. Significant in-plane and through-plane motion and the small width of the RV wall limits the use of tissue characterization in this ventricle. In our study, T1 analysis of the RV was feasible with a region-based instead of a pixel-based approach, albeit with a 17% exclusion rate, and contours were only placed in the thickest regions of the myocardium therefore not providing an assessment of the entire RV free wall. Therefore, strain analysis provides an ideal alternative, and as demonstrated in our findings, peak strain indicated the onset of RV dysfunction. RV dysfunction can contribute to and is affected by many disease processes^41^. As patients who undergo defibrillation or cardioversion are likely to have one or multiple of these underlying diseases impacting the RV^42^, it is thus important to not narrow findings on only the LV and expand assessments to the RV as well.

### Translation to clinical settings

This was a translational study, which used an experimental healthy animal model to first demonstrate that electrical interventions lead to myocardial injury and that these acute changes in the heart can detected by non-invasive imaging techniques confirmed by histology. We applied the standard clinical mapping and strain sequences and analysis techniques that have been validated and implemented into clinical settings to investigate cardiovascular disease^4,5,17^. Thus, the implications from our imaging findings can be translated to human investigations. It is important to consider that these imaging techniques can be prone to changes due to other factors occurring in a peri-operative environment including saturation levels, and heart rate^43,44^. Particularly the relative myocardial water content alters T1 relaxation times and this is relevant to fluid management. Luetkens et al. investigated the impact of hydration or dehydration state on T1- and T2-relaxation times^45^. Here, the hydration status led to a significant change of T1 and T2 relaxation times. Administered water also has an influence on functional parameters. Cardiac function is primarily dependent on systolic and diastolic function as well as loading conditions, to specify preload and afterload. Burns et al. demonstrated that LV peak systolic strain rate and peak systolic strain are sensitive to acute changes in fluid load in human subjects^46^. Moreover, it was demonstrated in swine that high afterload due to an increase in blood pressure can impact myocardial blood flow and strain^15,47^. Since the animals of this study received fluids during the extensive scanning, relative changes of myocardial water content is a possible confounder. Nevertheless, blood pressure, haemoglobin and hematocrit levels did not change between the two timepoints, and fluid administration was not different between groups.

There are further considerations prior to translating these findings to a clinical setting. A cumulative energy of 1000 J was applied from 5 shocks. This is higher than applied in a typical cardioversion in a clinical setting but is still within a realistic treatment range. For example in a study of 101 patients undergoing elective external cardioversion, Lobo et al. reported the median shocks and cumulative dose delivered was 1 shock at 161 J, yet up to 6 shocks with a cumulative dose of 1668 J was provided in one instance^48^. Dose-dependent relationships would be important to investigate in the future, in addition to other variables such as pad position^49,50^. In non-responders to electric cardioversion one could investigate if there is a potential benefit to determine the best vector for the electric current using imaging exams, however other factors, i.e. the electric resistance of the tissue between defibrillator pads, may play a more important role that the actual vector. This study investigates healthy swine who were in normal sinus rhythm prior to the intervention. This allowed us to isolate the impact of the transthoracic shocks without the confounding effect of underlying injury from an arrhythmia. As a result, we also observed that transthoracic shocks worsened synchronicity of segmental contraction, as shown by an increased mechanical dispersion. In contrast to our study, in a clinical scenario, the patients receiving this intervention will have existing arrhythmia. Future clinical studies will need to investigate how electrical interventions impact myocardial tissue features and function in an already diseased heart and the net impact of cardioversion comparing the benefit of restoring sinus rhythm in comparison to the induced myocardial injury. While the alternatives are limited to avoiding this intervention, a better understanding of the regional myocardial tissue abnormalities and dysfunction potentially induced by electrical procedures and the variation in the onset of dysfunction between individuals may improve the understanding of myocardial recovery post-cardioversion. Additionally, investigation of the myocardial injury arising from the electrical pathway in patients with unsuccessful cardioversions could facilitate more effective pad placement. This study focused on the acute effects of acute injury within a 5 h timeframe after electric transthoracic shocks and its impact on regional function, however, we do not know how long the dysfunction endures or if these result in a permanent injury. Further studies in clinical populations are required to establish if transthoracic shocks result in permanent effects on the myocardium.

## Conclusion

While serial transthoracic shocks in a healthy swine model led to a global attenuation in biventricular peak strain, the acute development of regional diastolic dysfunction was associated with the onset of colocalized myocardial injury. Future studies are warranted to assess these effects of electrical interventions in clinical settings.

## Data Availability

The datasets generated during and/or analysed during the current study are available from the corresponding author on reasonable request.
